# Congenital hepatic fibrosis in an adult female combined with Von Meyenburg complex and autosomal dominant polycystic kidney disease: a case report

**DOI:** 10.3389/fgstr.2026.1876545

**Published:** 2026-07-24

**Authors:** Xinrui Ren, Yu Cui, Jiayi Song, Yiwen Tang, Xiaoying Xie, Wanchun Zhu, Zhuo Yu, Lingying Huang, Yueqiu Gao

**Affiliations:** 1 Department of Liver Diseases, Shuguang Hospital Affiliated to Shanghai University of Traditional Chinese Medicine, Shanghai, China; 2 Laboratory of Cellular Immunity, Shuguang Hospital Affiliated to Shanghai University of Traditional Chinese Medicine, Shanghai, China; 3 School of Traditional Chinese Medicine, Shanghai University of Traditional Chinese Medicine, Shanghai, China; 4 Department of Hepatic Oncology, Zhongshan Hospital, Fudan University, Shanghai, China

**Keywords:** autosomal dominant polycystic kidney disease, congenital hepatic fibrosis, exome sequencing, PKD1, von Meyenburg complex

## Abstract

**Background:**

Congenital hepatic fibrosis (CHF) is a rare disease associated with the polycystic kidney gene, and the initial symptoms are often associated with portal hypertension, such as splenomegaly and esophageal varices. While the basic clinical features and pathogenesis of isolated CHF have been thoroughly studied, patients frequently present variable combined phenotypes including autosomal dominant polycystic kidney disease (ADPKD) and Von Meyenburg complex (VMC). The pathogenic mechanisms driving these composite phenotypes require further exploration.

**Methods and results:**

We report a case of an adult female patient who was admitted due to unexplained upper gastrointestinal bleeding. Upon admission, the patient was diagnosed with congenital hepatic fibrosis in conjunction with VMC and ADPKD, based on findings from liver puncture biopsy, exome sequencing (ES), biochemical tests, and imaging examinations.

**Conclusions:**

The PKD1 gene is the causative gene for this patient’s CHF combined with VMC and ADPKD.

## Introduction

Congenital hepatic fibrosis (CHF) is a rare autosomal recessive disorder associated with ductal plate malformation (DPM). Its pathological features are mainly bile duct plate malformation and fibrosis in the portal area, accompanied by changes related to portal hypertension, but the structure of hepatocytes and hepatic lobules remains basically normal. In 1956, Parker first mentioned abnormal fibrotic changes of the liver in his autopsy report, and in 1961, Kerr first named it as “congenital hepatic fibrosis”. The incidence and prevalence of the disease are not clear, and the literature has estimated its prevalence to be approximately 1/10,000 to 1/20,000 (1–4).

CHF is classically considered a feature of autosomal recessive polycystic kidney disease (ARPKD), where approximately 50% of cases present with hepatic involvement. However, CHF is rarely reported in association with ADPKD, a condition more commonly known for causing multiple hepatic cysts rather than diffuse fibrosis. In recent decades, isolated cases of CHF secondary to ADPKD, particularly in children with CHF, have been described in the literature, but reports of concurrent CHF, ADPKD, and Von Meyenburg complex (VMC) in adult patients remain extremely scarce (5, 6).

We report a rare case of congenital hepatic fibrosis (CHF) concurrent with VMC and ADPKD, with early clinical manifestations presenting in adolescence. The diagnosis was confirmed by medical imaging, liver biopsy, and genetic testing. Furthermore, we discuss the pathogenesis underlying CHF, fibrocystic liver disease (FLD), and hepatorenal fibrocystic disease (HRFCD) in light of this rare case.

## Case report

A 31-year-old woman underwent splenectomy at a local hospital in Suzhou at the age of 16 due to splenomegaly, and details of the procedure and underlying etiology were not available in her medical records. Surgical portosystemic shunt was not considered for this patient. Following her splenectomy, she failed to attend regular hospital follow-up visits, leading to long-term loss of standardized monitoring of portal hypertension and varices. Without continuous clinical assessment, multidisciplinary evaluation for shunt surgery was never initiated. Her father was healthy, her mother had PKD, and she had no siblings. In addition, she denied any history of chronic medical diseases, alcohol consumption, smoking, recent drug use, or infectious diseases such as hepatitis B. Five days prior to admission, she experienced black stool without any identifiable triggers, approximately 200 mL per day. There was no vomiting of blood, fever, or abdominal pain, so she did not pay particular attention to these symptoms. One day before admission, the patient developed dizziness and subsequently presented to the emergency department.

**Figure 1:**
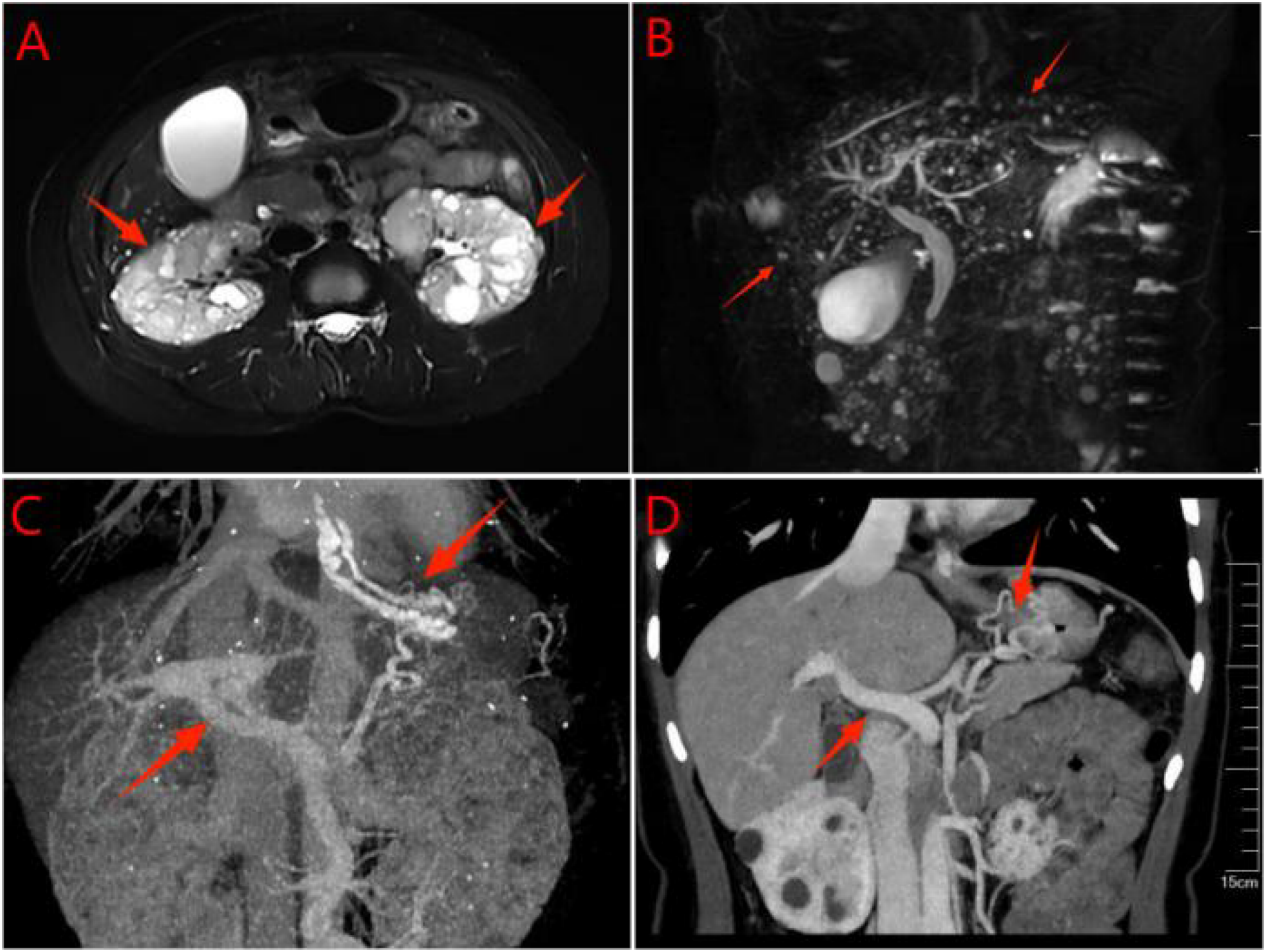
**(A)** Upper abdomen enhanced MRI (axial view, T2WI). The red arrows point to bilateral renal multiple nodular hyperintense lesions, confirming the feature of ADPKD. **(B)** Upper abdomen enhanced MRI (coronal view). The red arrows indicate intrahepatic cystic lesions presenting typical “starry sky” pattern without communication with the biliary tract, consistent with VMC. **(C, D)** Portal vein CTV (**(C)** coronal maximum intensity projection; **(D)** sagittal reformation). The red arrows mark dilated portal trunk (diameter ~17.5 mm) and obvious esophagogastric fundal varices caused by portal hypertension respectively.

Upper gastrointestinal endoscopy revealed Grade III esophageal varices involving the distal esophagus, with positive red color signs. Laboratory test results indicated abnormalities: erythrocyte count of 2.46 × 10^12/L, hemoglobin level of 68 g/L, alanine transaminase (ALT) of 62.89 U/L, γ-glutamyl transferase (γ-GT) of 111 U/L, and a fecal occult blood test result of (+++). Consequently, she was admitted to the hospital for further investigation due to “black stools”. Emergency gastroscopy was only performed for rapid etiological screening of acute upper gastrointestinal bleeding, and no active ruptured bleeding was observed. After comprehensive preoperative evaluation following admission, a second elective gastroscopy was scheduled to grade varices thoroughly and deliver prophylactic endoscopic hemostasis. Admission physical examination: No obvious jaundice of the skin and mucous membranes, no ecchymoses or petechiae. No superficial lymphadenopathy was palpable throughout the body. Spider angiomas and palmar erythema were absent. Sclerae were anicteric, and conjunctivae showed no congestion or edema. The abdomen was flat and soft. An old well-healed surgical scar approximately 8 cm in length was visible in the left upper abdomen. There was no abdominal tenderness, rebound tenderness, or muscular guarding. McBurney’s sign and Murphy’s sign were negative. The liver was not palpable below the costal margin, and hepatic percussion tenderness was absent. Shifting dullness was negative. Bowel sounds were normal without hyperactivity. Renal percussion tenderness was negative, and there was no bilateral lower limb edema. After admission, the patient underwent endoscopic esophageal variceal ligation and sclerotherapy, with no evidence of active postoperative bleeding. To further elucidate the patient’s level of liver fibrosis and the etiology of portal hypertension, a series of relevant examinations were conducted. The contrast-enhanced MRI of the upper abdomen revealed hepatic morphological abnormalities, elevated hepatic stiffness (elasticity value, 3.21–3.43 kPa corresponding to fibrosis stage F2 according to established diagnostic criteria) (7), and VMC (**Figures 1A, B**). Additionally, multiple bilateral renal cysts were observed. Portal vein computed tomography venography (CTV) demonstrated findings consistent with portal hypertension, including main portal vein dilatation to approximately 17.5 mm(**Figures 1C, D**). Laboratory investigations ruled out viral liver disease, autoimmune liver disease, alcoholic liver disease, drug-induced liver disease, schistosomal liver disease, and Wilson’s disease (**Table 1**). Ultrasound-guided percutaneous liver biopsy was performed; targeted sampling of Von Meyenburg complex lesions was not conducted.

**Table 1:** Baseline laboratory parameters of the patient after admission.

Category	Parameter	Value	Normal range
Blood routine			
	WBC in ×10^12^/L	12.71 ↑	3.69-9.16
	RBC in ×10^12^/L	2.39 ↓	3.68-5.13
	Hb in g/L	66 ↓	113-151
	PLT in ×10^12^/L	477 ↑	101-320
	CRP in mg/L	0.83	0.00-8.00
Liver function			
	TB in umol/L	9.5	0-23
	ALT in U/L	27	7-40
	AST in U/L	18	13-35
	ALP in U/L	98	35-100
	γ-GT in U/L	81 ↑	7-45U/L
	ALB in g/L	33.3	40-55
	TP in g/L	57 ↓	65-85
Kidney function			
	Scr in µmol/L	59	41-73
	eGFR in mL (min*1.73m^2^)	104.92	>90
	UA in µmol/L	293	155-357
Immune antibodies			
	ANA	(-)	N/A
	ENA	(-)	N/A
	Antimitochondrial	(-)	N/A
	Anti-smooth muscle	(-)	N/A
	Anti-liver-kidney microsomal antigen-1	(-)	N/A
	Anti-liver cytosol-1	(-)	N/A
	Anti-SSA	(-)	N/A
	Anti-SSB	(-)	N/A
	Anti-RO52	(-)	N/A
	IgG in g/l	11.5	7.2-16.9
	IgA in g/l	1.58	0.69-3.82
	IgM in g/l	0.96	0.63-2.8
	CP in mg/dl	33.44	22-58
Virological indicators			
	Hepatitis A virus	(-)	N/A
	Hepatitis B virus	(-)	N/A
	Hepatitis C virus	(-)	N/A
	Hepatitis D virus	(-)	N/A
	Hepatitis E virus	(-)	N/A
	Cytomegalovirus	(-)	N/A
	EB virus	(-)	N/A
	Syphilis	(-)	N/A
	Human immunodeficiency virus	(-)	N/A
Coagulation functions			
	PT in s	12.7	10.4-12.7
	D-Dimer in ug/mL	1.36 ↑	0-0.55

**Figure 2:**
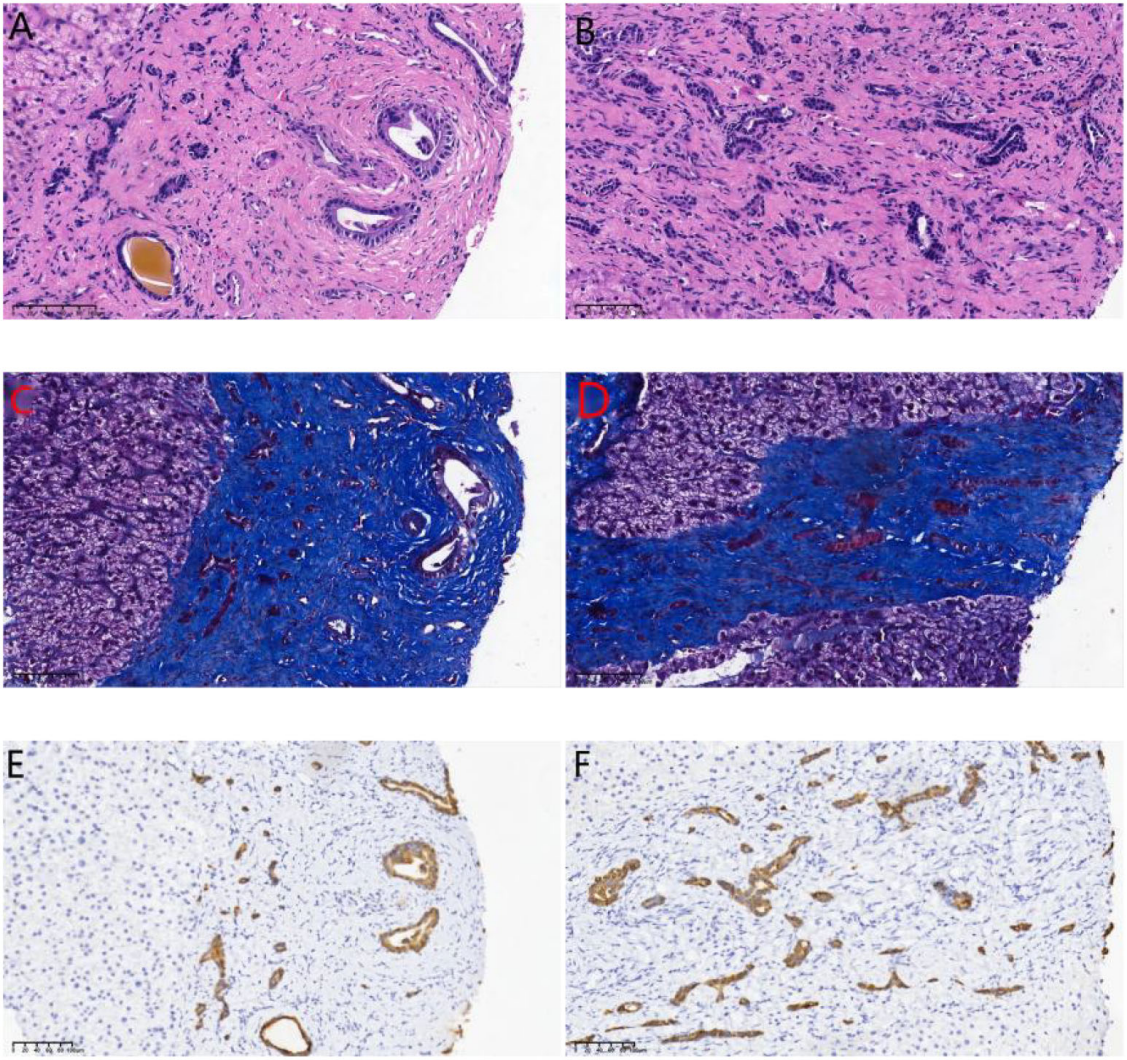
Histopathology of liver. **(A, B)** Histological examination reveals marked portal fibrosis with proliferated bile ducts demonstrating focal architectural distortion and associated bile stasis (H&E stain X500). **(C, D)** Prominent collagen deposition in portal tracts, consistent with progressive fibrogenesis (Masson stain X500). **(E, F)** Immunohistochemical staining demonstrates marked ductular proliferation in the portal tracts (Immunohistochemical CK19 X500).

**Figure 3:**
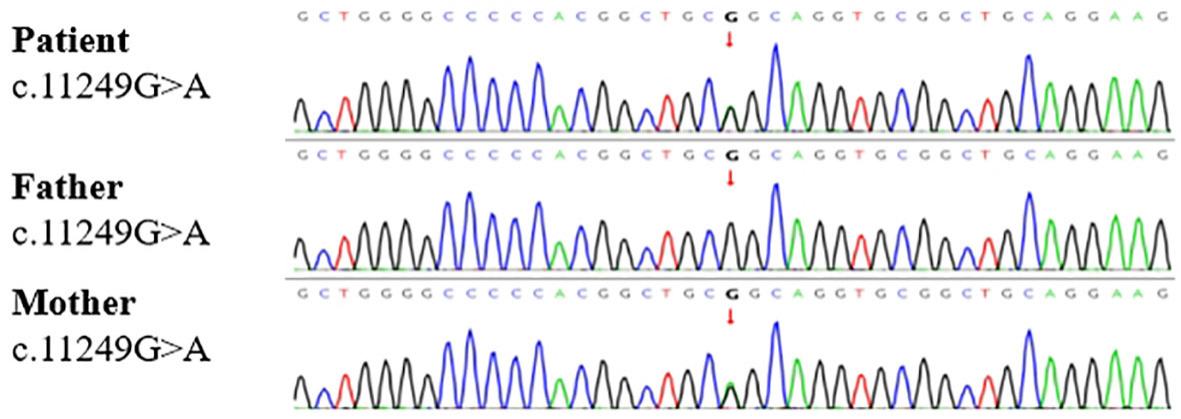
Sequencing results of the PKD1 gene. The PKD1 gene presents a heterozygous missense variant (C11249G>A: p.R3750Q), which is of maternal origin. In the coding sequence of the gene, the base at position 11249 was mutated from guanine (G) to adenine (A), and this base substitution resulted in the substitution of arginine (Arginine, R) for glutamine (Glutamine, Q) at position 3750.

Routine hematoxylin-eosin (HE) staining was applied for pathological examination in this study, and no special molecular pathological tests were performed. The pathological analysis (**Figures 2A–F**) demonstrated preserved lobular architecture with approximately 12 portal tracts. Hepatocytes within the lobules exhibited focal cytoplasmic rarefaction and scattered cytolytic necrosis, without evidence of hepatocellular steatosis. The confluent regions displayed mild chronic inflammatory cell infiltration, with focal areas of prominent fibrosis. Additionally, hyperplastic small bile ducts with architectural distortion and occasional bile plugs were observed, accompanied by reactive vascular proliferation. These characteristic biliary and fibrotic changes represent DPM, which is defined as retention of abnormal embryonic bile ducts in primitive ductal plate architecture, accompanied by aberrant biliary radicle dilatation and hepatic fibrosis. The final pathological diagnosis was congenital hepatic fibrosis secondary to ductal plate malformation. The patient and her parents underwent ES testing, which showed that a heterozygous missense variant was detected in the coding region of the patient’s PKD1 gene: C11249G>A: p.R3750Q, which was derived from her mother (**Figure 3**). Combined with the above findings, the patient was diagnosed with congenital hepatic fibrosis, VMC, and ADPKD. The patient was discharged after symptomatic supportive treatment and did not attend regular follow-up in our department thereafter.

## Discussion

**Figure 4:**
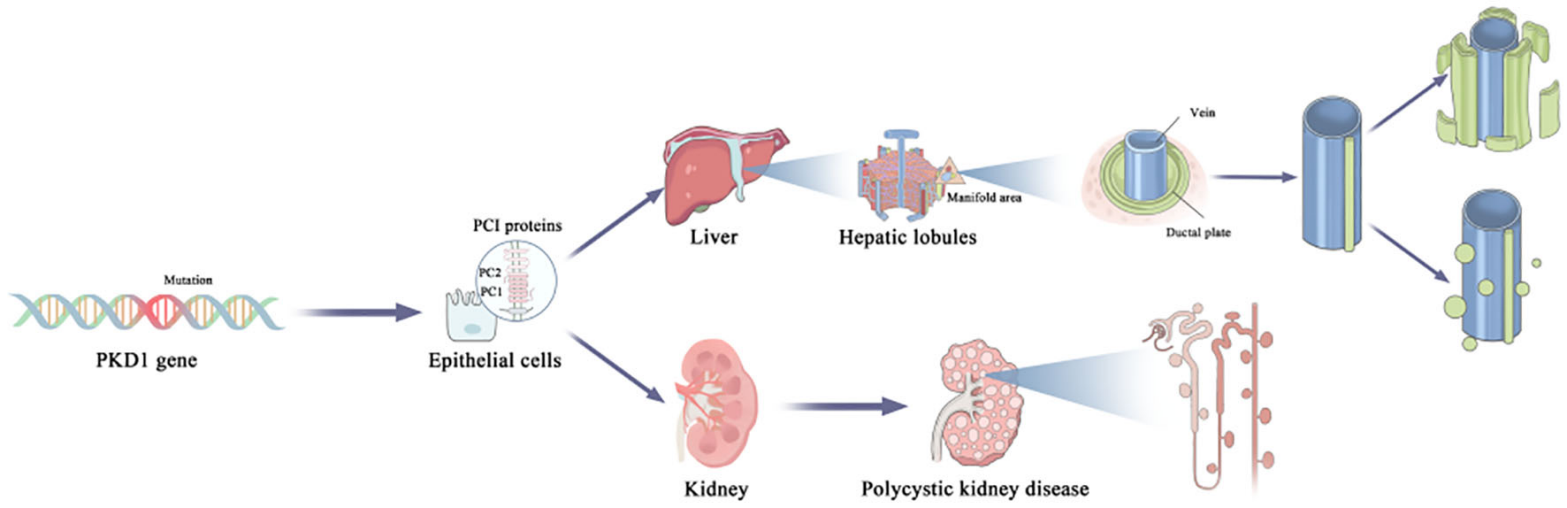
Pathogenetic mechanism diagram of the patient in this study: PKD1 gene encodes PC1. This protein is primarily expressed in the primary cilia of biliary and renal tubular epithelial cells. The normal development of both bile ducts and renal tubules relies on intact ciliary signaling. Mutations in the PKD1 gene lead to dysfunction of the cilia in biliary and renal tubular epithelial cells, which in turn cause malformations of the biliary plates, abnormal morphology and polarity of renal tubular cells, and ultimately result in CHF, VMC, and PKD.

Congenital hepatic fibrosis (CHF) is a rare inherited form of fibropolycystic liver disease (FLD). FLD encompasses several conditions, including CHF, Caroli disease, polycystic liver disease (PCLD), and VMC (1). The pathogenesis of FLD involves aberrant expression of embryonic developmental genes, chromosomal variants, and dysregulation of cell signaling pathways. Collectively, these factors contribute to progressive hepatic fibrosis and cystic degeneration of the liver parenchyma. The disease is characterized by progressive biliary epithelial hyperproliferation, biliary microdisturbances, and the formation of multiple dilated hepatic cysts (2). Ductal plate malformation represents a potential pathological mechanism underlying FLD. Ductal plate malformation (DPM) underlies FLD pathogenesis. Normally, primitive hepatoblasts around portal vein mesenchyme form a single-layered biliary plate by gestational week 8, which matures into a bilayered ductal plate via epithelial–mesenchymal crosstalk (**Figure 4**). Impaired remodeling causes interlobular bile duct dysplasia and intralobular biliary, forming VMC. Subsequent periductal inflammation, necrosis and sustained collagen deposition create periportal fibrous septa, and progress to CHF (3, 8). In the present case, the patient exhibited both CHF and VMC concurrently.

VMC, also known as intrahepatic biliary hamartoma, is a rare benign congenital liver lesion caused by maldevelopment of the embryonic ductal plate. The lesions specifically involve interlobular small bile ducts rather than intralobular bile ducts. Most patients are asymptomatic, and the disease is incidentally detected during imaging examinations (9). Ultrasonography and CT lack specific manifestations for VMC, while typical cases exhibit characteristic MRI features: lesions demonstrate hypointensity on T1-weighted images and marked hyperintensity on T2-weighted images, presenting a classic “starry sky” sign with well-defined borders, no communication with the native biliary tree, and no obvious enhancement after contrast administration (10, 11). The enhanced upper abdominal MRI of the patient in this study revealed the typical intrahepatic “starry sky” sign, which provided crucial imaging evidence for the diagnosis of VMC. Differential diagnosis of VMC mainly includes Caroli disease and polycystic liver disease. In Caroli disease, dilated bile ducts communicate with the biliary system and show linear band-like enhancement within cystic dilated bile ducts. Polycystic liver disease manifests as scattered round or oval cystic lesions with smooth margins, which differ distinctly from VMC characterized by irregular lesions diffusely distributed along the biliary tree (9). Clinically, high-resolution MRI can establish a definitive diagnosis for typical VMC. However, percutaneous liver core needle biopsy remains the gold standard for atypical lesions that cannot rule out malignant diseases such as hepatic metastasis and intrahepatic cholangiocarcinoma. Histopathological examination combined with immunohistochemistry can confirm the nature of lesions and exclude malignancy, offering decisive evidence for precise clinical management (11, 12).

Biliary plate malformations associated with fibrocystic liver disease arise from structural and functional defects in the primary cilia of bile duct cells. These malformations are part of a spectrum of developmental disorders resulting from abnormal ciliogenesis and are classified as ciliopathies (2). Cellular cilia are divided into motor and non-motor cilia (primary cilia). Motile cilia are expressed mainly in respiratory cells, tubal cells, and ventricular epithelial cells and are involved in regulating the movement of fluid across epithelial surfaces. Dysfunction of motor cilia causes diseases such as bronchiectasis, visceral inversion, and infertility. Primary cilia are sensory organelles expressed by most polarized eukaryotic cells, such as renal tubular epithelial cells as well as cholangiocytes. They are located on basal bodies (centrioles) and extend outward from the cell surface to act as signaling sensors between extracellular fluids (urine, bile, etc.) and the intracellular environment. Upon entering the cell cycle, non-motile cilia contain a group of proteins (including polycystins, fibrocystins, etc.) that mediate cell-to-cell and intercellular matrix interactions that are essential for tissue development, regeneration, repair, and homeostasis *in vivo*. Dysfunctional cilia disrupt cellular signaling pathways, thereby impairing organogenesis and physiological functions. Disorders arising from ciliary defects are collectively referred to as ciliopathies (5, 13). Ciliopathies can impact the functionality of nearly all organs, with a particular emphasis on the liver and kidneys. Hepatic ciliary dysfunction leads to hyperproliferation of cholangiocytes, induces DPM, and ultimately progresses to FLD (14, 15). Furthermore, ciliary dysfunction in renal tubular epithelial cells disrupts urinary flow mechanosensation, promoting cystogenesis and nephromegaly—hallmark pathological features of Polycystic Kidney Disease (PKD). This condition encompasses both Autosomal Dominant Polycystic Kidney Disease (ADPKD) and autosomal recessive polycystic kidney disease (ARPKD) (16). Current studies indicate that approximately 40% of patients with ciliopathies also present with concomitant hepatorenal fibrocystic disease (HRFCD), leading to its nosological classification as HRFCD (2). Clinically distinct ciliopathy entities manifest pleiotropic renal and hepatic pathologies, constituting a phenotypic continuum from non-syndromic fibrocystic disease to multisystem ciliopathies — including Meckel-Gruber syndrome (MKS), Bardet-Biedl syndrome (BBS), Joubert syndrome (JBTS), Senior-Loken syndrome (SLSN), COACH syndrome, and oral-facial-digital syndrome (OFDS). They can present with a range of symptoms in addition to CHF, including ataxia, developmental delay, oculomotor respiratory abnormalities, intellectual disability, various retinal defects, abnormal facial morphology, and polydactyly (17, 18). Notably, this case exhibited non-syndromic HRFCD — pathologically defined as fibrocystic liver disease with congenital hepatic fibrosis and ductal plate malformations, concomitant with autosomal dominant polycystic kidney disease — while lacking canonical syndromic features.

CHF rarely occurs concomitantly with ADPKD, while polycystic liver disease (PLD) represents the most common extra-renal cystic manifestation in patients with ADPKD (19). Approximately 77% of ADPKD patients develop hepatic cysts by the age of 60. PLD arises from progressive accumulation and dilation of cysts derived from biliary epithelium, and the vast majority of clinical manifestations stem from multiple hepatic cysts. Massive hepatomegaly secondary to cyst expansion compresses the esophagus and diaphragm, triggering systemic complaints including gastroesophageal reflux and dyspnea; overt liver-related adverse manifestations generally emerge only in the advanced stage of the disease (15, 19). Distinct from the typical phenotype of conventional PLD, the proband in our study presented with concurrent CHF and VMC, constituting an extremely rare combined fibrocystic hepatic lesion. We systematically summarized eight case reports published between 2016 and 2026 that documented ADPKD complicated by diverse hepatic fibrocystic disorders (**Table 2**), enrolling a total of 9 patients (5 females and 4 males) with an age range of onset from 25 to 63 years. The classification of hepatic fibrocystic lesions among these patients was as follows: 5 cases of isolated PLD and 3 cases of CHF. Associated concomitant manifestations included hepatomegaly (3 cases), Caroli syndrome (1 case), hepatic venous outflow obstruction (HVOO) (1 case), and Budd-Chiari syndrome (1 case). Only 3 of the 9 patients underwent genetic testing: two patients with ADPKD combined with CHF harbored missense variants in the PKD1 gene, and one patient with ADPKD complicated by Caroli syndrome carried a heterozygous deletion variant of PKD1. The proband of the present study suffered from ADPKD concurrent with both CHF and VMC. Whole-exome sequencing was performed on this patient, which identified a novel heterozygous missense variant in the PKD1 gene that has not been previously documented in the existing literature.

**Table 2:** Summary of previously reported cases of ADPKD combined with diverse FLD.

Case reference	Age	Gender	Family history	Liver presentation	Other manifestation	Genetic finding	Treatment strategies	Clinical course & final outcome
Fonseca et al. (19)	43 years	Female	Positive family history of liver disease	Hepatomegaly, PLD	Systemic portal hypertension	No genetic testing performed	Combined liver-kidney transplantation after 7 months of hemodialysis	Discharged 7 days after surgery
Awad et al. (20)	58 years	Male	His son was diagnosed with PKD	Massive PLD replacing most hepatic parenchyma, decompensated cirrhosis, hepatic venous outflow obstruction (HVOO)	End-stage renal disease, intestinal obstruction, hypertension, hyperlipidemia, gastroesophageal reflux disease	No genetic testing performed	Elective bilateral nephrectomy after 4 years of hemodialysis; concurrent primary repair for serosal intestinal injury and emergency splenectomy due to splenic laceration	Died of complications of liver failure and fungemia several days after transfer to transplantation center
Jiang et al. (Case 1) (21)	25 years	Female	Not mentioned	CHF, cirrhosis; abdominal ultrasound showed biliary dysplasia, hepatic fibrosis, portal vein thrombosis, portal hypertension with collateral circulation	Splenomegaly	Two heterozygous missense variants in PKD1: c.7670A>G (p.Asp2557Gly) in exon 19, c.5494G>A (p.Gly1832Ser) in exon 15	Splenectomy for severe anemia, combined with symptomatic conservative management	Discharged with relieved symptoms, survived with 2-year follow-up
Jiang et al. (Case 2) (21)	25 years	Male	Family history of HBV-related cirrhosis; no personal viral hepatitis infection	CHF, hepatomegaly	Splenomegaly	No genetic testing performed	Orthotopic liver transplantation	Discharged with improved symptoms, survived with 2-year follow-up
Akuzawa et al. (22)	54 years	Female	Father had a history of hypertension, cerebral infarction and myocardial infarction	PLD	Hypertension, iron-deficiency anemia, abdominal wall hernia, intestinal obstruction	No genetic testing performed	Hepatic cyst fenestration and right hepatectomy for massive hepatomegaly	Deceased after surgery
Mizuta et al. (23)	37 years	Male	Father diagnosed with ADPKD	PLD, hepatomegaly	Chronic renal failure	No genetic testing performed	Transarterial embolization (TAE) for hepatic cysts, with unsatisfactory efficacy and progressive hepatomegaly; liver transplantation planned; maintenance hemodialysis for progressive renal failure	Developed progressive liver enlargement leading to fatal liver failure
de Menezes Neves et al. (24)	50 years	Female	Two maternal aunts diagnosed with ADPKD	PLD, cyst-induced hepatic vein stenosis complicated with Budd-Chiari syndrome and portal hypertension	End-stage renal disease, ascites	No genetic testing performed	Combined liver-kidney transplantation; long-term maintenance with mycophenolate sodium 360 mg qd, prednisone 5 mg qd, tacrolimus 3 mg bid	At 1-year postoperative follow-up, hepatic and renal function remained normal, abdominal volume decreased and quality of life significantly improved
Khudhair et al. (25)	63 years	Male	Father, paternal uncle and brother all diagnosed with polycystic kidney disease	MRCP confirmed Caroli syndrome with segmental cystic dilatation of proximal bile ducts in both hepatic lobes; shear wave elastography indicated mild hepatic fibrosis	No other comorbidities	Heterozygous deletion spanning exons 32–34 of PKD1 (NM_001009944.3: c.10167 + 11_10499 + 1289del)	Broad-spectrum antibiotics and intravenous fluid infusion for fever and abdominal pain	No recurrent cholangitis or jaundice thereafter; regular follow-up in nephrology department
Liu et al. (26)	31 years	Female	Father suffered from hepatosplenomegaly and bilateral multiple renal cysts; elder brother underwent splenectomy for variceal bleeding induced by portal hypertension 5 years ago, with bilateral renal cysts	CHF	Splenomegaly	Compound heterozygous variants in PKD1: paternally inherited c.8296T>C (p.Ser2766Pro) and maternally inherited c.9653G>C (p.Trp3218Ser)	Endoscopic band ligation and sclerotherapy for gastroesophageal varices	Markedly relieved clinical symptoms after 18 months of standardized treatment and follow-up

The causative genes associated with hepatic and renal fibrocystic disease are complex and variable, and the most common mutated genes in internationally reported cases are PKHD1, PKD1, PKD2, and NPHP1, etc. In our case, the patient was detected with the PKD1 gene. The PKD1 gene is located on human chromosome 16p13.3 and has 46 exons. PKD1 encodes polycystin 1 (PC1) (27). This protein is mainly expressed on the primary cilia of the bile duct and renal tubular epithelial cells. Normal development of both bile ducts and renal tubules is dependent on intact cilium signaling. PKD1 variants lead to dysfunction of bile duct epithelium and renal tubular cilia, which further causes biliary plate malformations, abnormalities in renal tubular cell morphology and polarity, leading to FLD and renal cyst formation (**Figure 4**). Variants in the PKD1 gene are varied and include shortening, missense, splice-site, deletion, and nonsense variants (26). In this case, the patient and her parents underwent Exome sequencing, which showed that one heterozygous missense variant was detected in the coding region of the PKD1 gene: C11249G>A: p.R3750Q, which originated from the mother. Variant sites for the PKD1 gene variants have been reported in the literature as c.7670A>G, p. Asp2557Gly; c.5494G>A, sp.Gly1832Ser; c.6730 -673delp.(Ser 2244Hisfs*17); c.8296T>C (Ser2766Pro), and others (21, 26, 28). Further investigation indicated that the patient’s mother had a history of polycystic kidney disease (PKD), implying that both the mother’s and daughter’s PKD may be attributed to a Variant in the PKD1 gene. Of note, the missense Variant c.11249G>A (p.R3750Q) identified in this study has not been previously reported in the literature. According to the ACMG/AMP 2015 variant classification criteria, this variant was classified as likely pathogenic based on multiple lines of evidence, including its extreme rarity in the general population (PM2_Supporting), previous reports in PKD cases (PS4), deleterious predictions by in silico tools with a REVEL score of 0.839 (PP3_Moderate), and high consistency with the phenotypic spectrum of PKD1-related hepatorenal fibrocystic disease (PP4). Therefore, this novel missense Variant is considered to have a deleterious effect and is likely responsible for the disease phenotype in this patient. The patient’s mother is 56 years old. Unfortunately, she declined all clinical examinations and auxiliary investigations apart from exome sequencing, including liver imaging and liver biopsy. Given that the patient’s mother currently exhibits no clinical symptoms of liver disease and the patient had CHF and VMC, it was hypothesized that the patient may harbor Variants in other modifier genes. Accumulating genetic evidence has confirmed that modifier genes exert critical regulatory effects on the phenotypic heterogeneity of autosomal dominant polycystic kidney disease (ADPKD), which accounts for the substantial variability in the severity of renal and hepatic lesions both within families and across distinct pedigrees carrying identical pathogenic PKD1/PKD2 variants (29). According to the literature, PKHD1 and other ciliopathy-related genes (e.g., RPGRIP1L) (30) may act as potential modifiers. However, the complete molecular interaction network between pathogenic mutations and modifier loci remains incompletely elucidated, and standardized clinical risk stratification models incorporating modifier genes are currently lacking. Accordingly, systematically dissecting the functions and prognostic predictive value of modifier genes represents a promising translational research priority in this field (29).Furthermore, genetic counseling is an indispensable component of standardized diagnosis and management for the proband in this case. Pre- and post-test genetic counseling enables systematic interpretation of the novel heterozygous missense PKD1 variant identified via whole-exome sequencing in this patient, clarifies the autosomal dominant inheritance pattern and the 50% recurrence risk for offspring, guides imaging screening for high-risk family members, and delivers individualized reproductive guidance including prenatal diagnosis and preimplantation genetic testing (PGT). Meanwhile, genetic counseling alleviates psychological distress such as guilt and anxiety caused by disease heritability, facilitates genetic risk evaluation for living-related kidney donation, and comprehensively optimizes the long-term integrated management strategy for ADPKD patients complicated with complex fibrocystic hepatorenal lesions (31).

Currently, there is no specific treatment that can stop or reverse the progression of hepatic and renal fibrocystic disease, and the clinical treatment is mainly aimed at liver and kidney related complications. Our patient was diagnosed with hepatic and renal fibrocystic disease, which was specifically manifested as CHF, VMC, and ADPKD. The management of CHF in this patient was centered around symptomatic relief for various complications predominantly caused by portal hypertension. Internal treatments included hemostatic measures, oral β-blockers, endoscopic interventions for esophagogastric fundus varices, among others. Surgical options mainly consisted of shunt procedures and flow interruption techniques. Currently, liver transplantation remains the only definitive cure for patients suffering from end-stage CHF (1). In addition, patients with ADPKD mainly present with increasing size and number of renal cysts, which destroys normal renal structure and function, and about 50% of patients will progress to end-stage renal disease (ESRD) at the age of 60 years. It may manifest as low back and abdominal pain, hematuria, kidney stones, urinary tract infection, proteinuria and hypertension. Current treatment is based on symptomatic management (32). Tertiary management is implemented according to the KDIGO guidelines (32): strict salt restriction (<5 g/d), sodium restriction (<2 g/d), control of protein intake (0.8 g/kg/d), smoking cessation and alcohol restriction, avoidance of nephrotoxic substances and collision sports, and other basic interventions, the use of tolvaptan to slow down the disease progression (33), and renal transplantation in end-stage.

In recent years, molecular biological studies have clarified that primary ciliary dysfunction constitutes the core pathogenic mechanism of autosomal dominant polycystic kidney disease (ADPKD). Based on this mechanism, a variety of novel targeted agents and innovative therapeutic modalities have been developed, beyond the previously mentioned mTOR inhibitors (e.g., everolimus) and CFTR modulators (the lumacaftor/ivacaftor combination), opening new avenues for precise individualized treatment (34).First, metabolic pathway-targeted drugs represent a prominent research hotspot. GLP−1 receptor agonists (such as semaglutide) suppress cyst proliferation via reprogramming glycolysis and ameliorating mitochondrial function; sodium-glucose cotransporter 2 (SGLT2) inhibitors alleviate glomerular hyperfiltration and interstitial inflammation, exerting synergistic renoprotective effects when combined with tolvaptan (35, 36).Second, somatostatin analogues (octreotide, lanreotide) serve as first-line targeted agents for patients with ADPKD complicated by severe polycystic liver disease. These agents inhibit cholangiocyte proliferation and reduce hepatic cyst volume by lowering intracellular cAMP levels in biliary epithelial cells, which confers critical clinical management value for the hepatic fibrocystic disorders discussed in the present study (37).Third, epigenetic and gene-editing innovative therapies remain in the preclinical research stage. The highly selective HDAC6 inhibitor GV−001 exhibits multiple beneficial activities including cyst-suppressive, anti-inflammatory and anti-fibrotic effects; anti-miR−17 oligonucleotides restore the expression of polycystin proteins; CRISPR base-editing technology can directly correct pathogenic variants in the PKD1 gene and reverse hepatic and renal cystic lesions in animal models (38–40).Additionally, metformin, an AMPK activator, demonstrates favorable anti-inflammatory and anti-proliferative efficacy in preclinical ADPKD models. Multiple large-scale randomized controlled trials are currently underway to validate its clinical value as an adjuvant therapeutic agent (41).

## Data Availability

The original contributions presented in the study are included in the article/supplementary material. Further inquiries can be directed to the corresponding author.
